# Resilience, relevance, remembering: history in the time of coronavirus

**DOI:** 10.5195/jmla.2020.986

**Published:** 2020-07-01

**Authors:** Stephen J. Greenberg

**Affiliations:** 1 stephen.greenberg@nih.gov, History Matters Editor and Section Head, Rare Books & Early Manuscripts, History of Medicine Division, National Library of Medicine, Bethesda, MD

## Abstract

In a time of unprecedented and rapid change, what are the roles of librarians and archivists in documenting the course of a pandemic?

**Figure:**
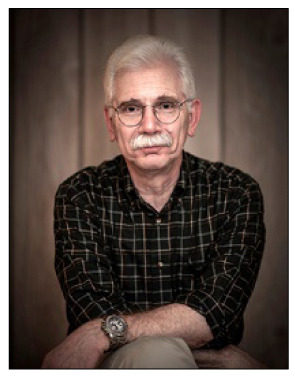
Stephen J. Greenberg, MSLS, PhD, AHIP

History, as I have quoted or paraphrased before in this column, is not what you think it is. History is what you remember. All other history defeats itself. Five years, ten years, fifty years from now, what will be remembered from what we are going through now? And how will that record be made?

In one important sense, none of this is new. Our species has seen pandemics before, and we have—more or less—muddled through. Newspapers and blogs helpfully provide lists and scorecards for a macabre top ten pandemics. It is easy to start with the famous ones: the Black Death of the fourteenth century, the 1918–1919 Flu (Figure 1), and HIV/AIDS. The more sophisticated and discerning student can consider the almost total destruction of the Native Peoples of North America by what we dismiss as “childhood” diseases that are now avoided by a simple shot or two, usually before twenty-four months of age. That particular destruction is especially hard to gauge, because in the absence of written records, we will never know how many died.

**Figure 1:**
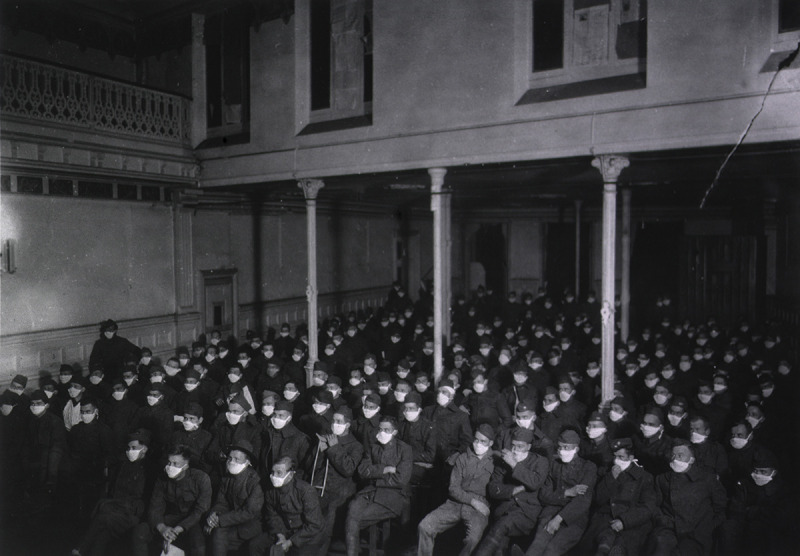
American soldiers in France, masked against influenza, c.1919

Courtesy US National Library of Medicine.

History depends on records, and there are many, many kinds of records. Some are from laboratories, some are clinical, some are statistical, and then there are those whose special value lies in the fact that they emerged from fields far distant from science. A colleague of mine, an epidemiologist with both a long clinical career and an abiding respect for history, has found himself sidelined for now with a non-COVID-related injury. To pass the time, he has been reading the *Decameron*, the fourteenth century set of tales written by Giovanni Boccaccio (1313–1375) during and immediately after the Black Death in Italy. The framing device for the stories (there are exactly 100 of them) is that a group of young aristocrats, 7 women and 3 men, have chosen to ride out the plague in Florence by escaping to a deserted villa in the countryside. For 10 days, they tell each other stories to while away the time. Let there be no mistake, this is not self-quarantining; they hope to hide from the contagion behind the walls of wealth and privilege. After the 10 days of storytelling, they return to Florence and their uncertain fates. Florence, a thriving city with a population of about 80,000, lost somewhere between 45% and 75% of its population to the plague in the years 1348 through 1351.

Many critical and literary sources tout the instant popularity and widespread influence of the work, and my colleague was both fascinated and mystified by these claims. After all, he wrote to me, what did “popularity” and “widely read” mean in the early Renaissance world, a century before Gutenberg and printing in Europe. And how does one measure influence, anyway?

At one level, the answers are simple enough. The use of such terms as “widely read” makes no sense at all in the fourteenth century. The “masses” were illiterate anyway, even if the books had been “widely” available, which they were not. Literacy for the masses was not seen as a desirable thing until after the Protestant Reformation, when folks were supposed to read the Bible for themselves (no priest required). Even using the word “published” in the fourteenth century is sketchy—too many anachronistic associations.

But there was already something resembling a book trade in handwritten books. So, when Boccaccio started distributing the stories that would later be collected as the *Decameron* (roughly 1349–1351, but presumably started earlier), he could hire professional copyists to make fair codex copies, and there were booksellers to sell and resell the books. Numbers are always hard to come by, but one scholar counted fifty-eight booksellers and sixty-eight copyists in Paris before 1300. Books also got handed around—often in pieces—with certain bits being more popular and, therefore, more often copied, like the story of that poor dope Grizelda. This would have been a court and courtier thing, not a university thing. After all, Boccaccio (and Dante, and Petrarch) chose to write in a Tuscan dialect of Italian, not Latin.

Influence was a bear to measure (still is!), but scholars do know that some folks read and praised Boccaccio highly in fashionable court circles. Petrarch (Francesco Petrarca, 1304–1374) was a big fan, and he was an indefatigable letter writer and traveler. Petrarch was also quite the arbiter of “popular” taste. There were also copycat works, at least in using the framing device of a random group thrown together and sharing stories for their edification and amusement. The most famous was *The Canterbury Tales*, written by Geoffrey Chaucer between 1387 and 1400.

The Great Plague of London (1665–1666) had its literary classic too, although it is an odd one: Daniel Defoe's *Journal of the Plague Year*. Its oddity lies in the fact that, although it is presented as an eyewitness account of the last great outbreak of bubonic plague in London, it was not published until 1722. Defoe was five years old in 1665. Modern scholars accept that Defoe based his hair-raising account on an uncle's actual journal entries, although those journals are not extant. Literary and historical scholars quibble about how to classify the work (fact or fiction? history or historical romance?), but there is no denying or dismissing its immediacy or power for the average reader. Defoe's readership was just that; literacy rates were high in England by 1722, printed books were widely and (relatively) cheaply available, and the population was accustomed to seeing epidemics discussed. The London City Council had been authorizing the distribution of printed bills of mortality for over a century, and the availability of such numbers over an extended period of time had allowed John Graunt and Sir William Petty to invent something like a statistically based epidemiology: Big Data in the seventeenth century.

There are other literary and historical classics that have their roots in epidemic and pandemic disease. Some—like *The Plague* (*La Peste*, 1947) by Albert Camus or *Love in the Time of Cholera* (*El amor en los tiempos del colera*, 1985) by Gabriel Garcia Marquez—are really parables or allegories rather than historical accounts. But their structures work because the reader already understands the background against which the events will play out. The disease is just as much a framing device as Boccaccio's ten aristocrats in their Florentine villa.

Strangely enough, the Pandemic Flu of 1918–1919, arguably the deadliest pandemic to date, has no particular literary or historical classic of its own. Perhaps the writing world simply could not come to terms with such a vast event. Or perhaps they were (and at some level still are) already numbed by the carnage of the First World War.

But our pandemic is ongoing. As librarians, archivists, and other varieties of information professionals, how are we to deal with that? Uniquely, this pandemic seems to have fostered its own information overload. There was certainly no dearth of reporting about Ebola, severe acute respiratory syndrome (SARS), Middle East respiratory syndrome (MERS), and all the others, but COVID and information about it seem to have burst upon the world with a swiftness that was absolutely shattering (the cutesy phrase “went viral” seems oddly inappropriate and tasteless here). There will be many blog posts, articles, books, and doctoral dissertations written about how various authorities responded to that flood of information, and it will be undeniable that the flood existed.

Some librarians and archivists were extremely quick off the mark. The National Library of Medicine used its “Global Health Events” web archive as a place to start. The archive was created in 2014 to record materials on the Ebola virus and, later, the Zika virus. The coverage was expanded to include COVID on January 30, 2020, when the World Health Organization declared the outbreak a “Public Health Emergency of International Concern.” The professional group known as Librarians, Archivists, and Museum Professionals in the History of the Health Sciences (LAMPHSS) has already begun a robust email discussion on how to proceed: what to collect, how to make material available, and how to best share information and resources. LAMPHHS's 2020 annual meeting will be virtual, but the work is already underway. Not a day goes by without some new plan, some new approach, some new shared endeavor to gather and preserve what we are painfully learning, bit by agonizing bit.

Therein rest the core questions: what do we collect; what will we remember? The Big Data are being captured, but what we will tell our grandchildren, and what will our children tell *their* grandchildren? How will we document elbow bumps, homemade face masks, and attempts to refashion continuous positive airway pressure (CPAP) machines into ventilators? Why, the future will wonder, was there an apparent worldwide crisis in the toilet paper supply chain? Who got to decide what professions were “essential?” Here is where the real heroes were to be found; not just the doctors and nurses and their support staff, but the delivery truck drivers and grocery clerks and all the other unsung professions. An oral history project on a grand scale will be needed to capture their stories, but we are not ready for that, not yet.

Closer to home, how essential are librarians, such those in hospital libraries who stay on the job to support their institutions? And what of those in public libraries? One kind of common sense says to send them home for their own safety and lock the doors. But what of their patrons? Not the ones who want to borrow a DVD for Friday night or that long novel they always meant to read, but the ones who depend on the library for their Internet access? When you have no computer or access of your own, how do you apply for unemployment benefits when the state offices are all closed?

All this must be remembered, or else we learn nothing. History doesn't repeat itself, wrote Mark Twain, but it does rhyme. We need to recognize those rhythms and patterns, since they will link us to the past and point us to the future. Only memory, proper historical memory, can help us recognize and record change. Some things will come back, and some things are already gone, perhaps for good. That is an historian's job—to remember how it was then and how we got to where we are now.

So, as we wrangle over the relative merits of Zoom versus Webex and plan our cautious afternoon walks with careful regard for the current standards of “social distancing” (Figure 2), we are more—far more—aware that we live in interesting times, and that History, with a capital “H,” is breathing down our necks. One day, there will be a cure or at least a treatment. One day, there will be a vaccine. One day, it will be “over.” We are all historians right now, and the task is to remember the beginning.

**Figure 2:**
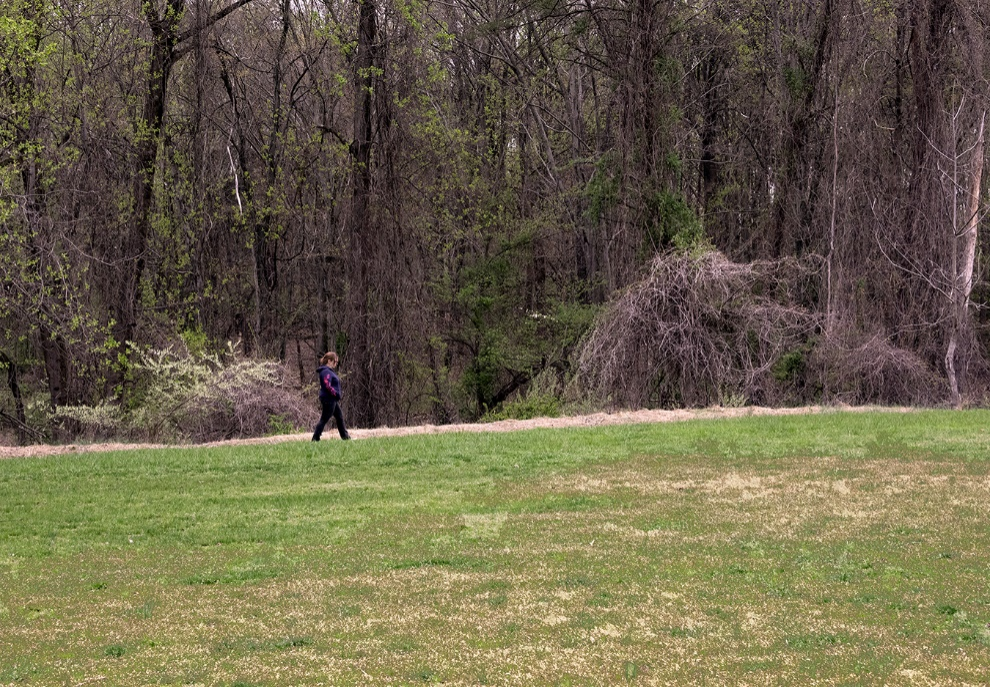
Social distancing in Maryland, 2020

Courtesy of the author.

I write these lines in mid-April; by the time they are published, it will be July. What will our History be then?

